# Rationale, Design, and Intervention Development of a Mobile Health–Led Primary Care Program for Management of Type 2 Diabetes in Rural Thailand: Protocol for a SMARThealth Diabetes Study

**DOI:** 10.2196/59266

**Published:** 2024-08-16

**Authors:** Methee Chanpitakkul, Devarsetty Praveen, Renu John, Arpita Ghosh, Salyaveth Lekagul, Malulee Kaewhiran, Kriang Tungsanga, Vivekanand Jha

**Affiliations:** 1 Bhumirajanagarindra Kidney Institute Hospital Bangkok Thailand; 2 The George Institute for Global Health Hyderabad India; 3 University of New South Wales Sydney Australia; 4 Prasanna School of Public Health Manipal Academy of Higher Education Manipal India; 5 Kamphaeng Phet Provincial Health Office Kamphaeng Phet Thailand; 6 Chulalongkorn University Bangkok Thailand; 7 School of Public Health Imperial College London United Kingdom

**Keywords:** health workforce, primary health care, diabetes, digital technology, Thailand, capacity building.

## Abstract

**Background:**

Noncommunicable diseases (NCDs), particularly diabetes and chronic kidney diseases, pose a significant health burden in Thailand, especially among socioeconomically disadvantaged populations. The existing primary health care system faces challenges in providing optimal care for NCDs due to inadequate primary care workforce. The SMARThealth program offers a technology-based solution to enhance NCD management through task-sharing among nonphysician health care workers.

**Objective:**

This study aims to adapt and implement the SMARThealth Diabetes program in rural Thailand to improve diabetes management. The main objectives are to (1) adapt, validate, and integrate the SMARThealth Diabetes program for improving the management of type 2 diabetes mellitus at the primary health care level; and (2) to determine the feasibility and acceptability of the SMARThealth Diabetes program in rural communities of Thailand.

**Methods:**

A pragmatic, type 2 hybrid effectiveness or implementation, parallel-group cluster randomized controlled trial of 12 months duration and involving 51 subdistrict health offices in rural communities of Kamphaeng Phet province, Thailand, will be conducted. The intervention arm will receive the SMARThealth Diabetes program, including workforce restructuring, clinical decision support system, and continuous performance monitoring, while the control arm will continue with usual practice. Data will be collected using the SMARThealth platform and will be stored on a server in Thailand. The primary outcome measure will be the change in mean hemoglobin A_1c_ (HbA_1c_) measured at randomization and 12 months from randomization between the intervention and control clusters. Secondary outcomes will include the difference in change in albuminuria status, estimated glomerular filtration rate, systolic blood pressure, and low-density lipoprotein cholesterol level. The analysis for change in HbA_1c_ between baseline and end of study will be performed using linear mixed models. Any imbalances between the 2 arms will be addressed by sensitivity analyses. Additionally, a mixed methods process evaluation will be conducted to assess the implementation process, that will include in-depth interviews and focus group discussions, in addition to the quantitative data collected during the implementation process. The qualitative data will be thematically analyzed to explore factors that promote or inhibit the implementation and maintenance of the program.

**Results:**

The data collection commenced in November 2022, and the results will be ready for publication by the first quarter of 2025. Effectiveness of the intervention package will be assessed by change in mean HbA_1c_ measures, and detailed feasibility, barriers, and enablers for the implementation of the intervention will be documented through a detailed process evaluation.

**Conclusions:**

The study protocol outlines a novel approach to enhancing diabetes management in rural Thailand through digital technology–based interventions that will facilitate task-sharing among health care workers. This can help inform future strategies for improving NCD care in low-resource settings globally.

**Trial Registration:**

Thai Clinical Trials Registry TCTR20200322006; https://www.thaiclinicaltrials.org/show/TCTR20200322006

**International Registered Report Identifier (IRRID):**

DERR1-10.2196/59266

## Introduction

### Background

South-East Asian countries (including Thailand) have been experiencing a steady rise in the burden of noncommunicable diseases (NCDs). The Global Burden of Disease Study 2015 reported diabetes and chronic kidney diseases (CKD) as Thailand's third and fifth leading causes of death [1]. According to the 5th Thai National Health Examination survey conducted in 2014 [2], Thailand has 4.63 million adults with diabetes (9.9% of the adult population), which is expected to grow to 5.2 million by 2035. In addition, 7 million adults in Thailand have prediabetes due to increasing childhood and adolescent obesity and is estimated that 25%-48% of people with diabetes will develop CKD [3]. According to the Microalbuminuria Prevalence Study survey in 2002 [4] across 10 Asian countries, it was found that 45% of the population in Thailand with diabetes had microalbuminaria and 15% had macroalbuminaria. Both conditions increase the risk for the development and progression of CKD, which will eventually lead to the need for dialysis or a kidney transplant. According to data from the Thai Renal Registry, diabetes was responsible for 34.6% cases of kidney failure in 2007 [5], increasing to 41.5% in 2020 [6]. Suboptimal treatment of diabetes leads to other macro- and microvascular complication such as cardiovascular disease, neuropathy, and retinopathy, all of which are associated with the use of more resources [7] and have grave economic and health impact.

The risk of suboptimal care and poor outcomes is worse among the socioeconomically disadvantaged. According to the World Bank reports, about 4.9 million people in Thailand live below poverty lines, with catastrophic health care cost being one of the factors contributing to relapse into poverty [8]. However, the launch of the universal health coverage program in Thailand that provided a comprehensive benefits package and zero copayment for health services was followed by a progressive decrease in the number of households (6% in 1996 to 2% in 2015) that experienced financial catastrophic events (defined as out-of-pocket payment for health exceeding 10% of household total consumption expenditure) due to health care [9].

Fortunately, the adverse outcomes, including the development of microvascular complications and progression to kidney failure can be prevented by optimal glycemic and blood pressure (BP) control and institution of effective interventions for CKD prevention. Healthy lifestyle (maintaining ideal body weight, physical activity, healthy diet, and smoking cessation), and pharmaceutical interventions including glycaemia management, BP control, use of renin-angiotensin system blockers, sodium-glucose cotransporter inhibitors, mineralocorticoid antagonists, and statins can substantially prevent the development of cardiovascular diseases and progression of kidney disease [10-12].

The key to early detection and prevention of renal and other complications in diabetes is the provision of effective and affordable primary care. However, major challenges undermine these efforts, especially among developing countries. For example, the size of the primary care workforce in developing countries is generally inadequate to meet the rising needs of giving care to those with diabetes and its complications. Maldistribution of health care workforce occurs in Thailand, similar to other developing countries. Due to the disproportionate doctor-population ratio, especially in the rural areas, there are not enough medical doctors to provide regular and optimum out-patient care to all patients with diabetes. The doctor-to-population ratio in Bangkok metropolitan area is as high as 1:515, whereas such ratio in the rural area is as low as 1:1723 to 1:2761 in the central and north-eastern regions, respectively [13]. In rural areas, it is commonly observed that those without serious complications could see a doctor in person only once or twice a year.

### Health System in Thailand

Under the government's universal health coverage policy implemented in 2002, all Thai citizens are eligible to receive essential health care services with support from public health funding [14]. The Ministry of Public Health (MOPH) is the country’s largest public-sector agency, controlling over two-thirds of all hospitals and beds. The MOPH divides the country into 12 health administrative regions exclusive of Bangkok metropolitan area. Altogether, there are 165 tertiary, quaternary, or specialty hospitals (300-1000 beds) at the regional and provincial levels, 743 secondary and tertiary care hospitals (10 to 250 beds) at a district level, and about 9800 subdistrict health offices (SDHOs) [15]. The SDHOs provide basic clinical care, as well as health promotion and primary disease prevention, to more than 44 million (80%) Thais living in rural areas [15]. Each SDHO employs 1-2 community nurses, 1-2 public health officers, and 1 dental therapist to care for 3000-6000 residents’ households. Though there is a well-connected system of patient referral from SDHO to its respective district hospital, most primary care services at SDHO are provided by its community nurses and public health officers.

In response to the growing burden of NCDs among the rural Thai population, the Thai MOPH launched the Thailand healthy lifestyle strategy 2018-2037 with the goal of improving care of diabetes and hypertension in public sector hospitals [16]. Each public hospital and SDHO must report the percentage of population screened for and found to have diabetes or hypertension and those having CKD. Though clinical practice guidelines have been developed, guideline-based clinical care is still far from reaching the targets. Only a minority of individuals with diabetes receiving continuous care could achieve recommended treatment targets for BP control (29.8%) and glycemic control (35%) [17].

Successful prevention initiatives after diabetes screening have been reported recently [14]. A cluster randomized study examining the effectiveness of integrated care on delaying the progression of stage 3-4 CKD in rural Thailand (ESCORT study) [18], evaluated integrated CKD care involving group counseling from a multidisciplinary team of district hospital staff and quarterly home visits by the community CKD care network (consisting of subdistrict health care officers and village health volunteers).  The intervention was able to slow the decline in estimated glomerular filtration rate (eGFR) over a 2- to 3-year period (difference 2.74, 95% CI 0.60-4.50 mL/min/1.73 m²; P=.009), showing the feasibility of using nonphysician workforce to augment primary care delivery and underscoring the need to develop effective strategies to deliver low-cost, evidence-based treatments for the prevention of diabetes and diabetic kidney disease that can be implemented in the primary health care systems [18]. In the subsequent prospective follow-up study, ESCORT II study [19], the integrated care model was extended to 5 district hospitals without a parallel control group. This expanded approach, which encompassed hospital multidisciplinary care and community home visits, resulted in a consistent mean eGFR decline of –0.92 mL/min/1.73 m²/year over the 36-month duration [19]. These findings further underscore the effectiveness of the integrated care model at the community level in effectively delaying the progression of CKD.

### SMARThealth

The SMARThealth program, based on a technology platform developed by the George Institute for Global Health, enables communities and health care providers to prevent and manage NCDs using guideline-based care, and continuous quality control [20]. This initiative is based on principles of “task-sharing,” in which routine clinical procedures are conducted by nonphysician health care workers, including village health volunteers, community nurses, and public health officers to increase access to quality health care and reduce costs. SMARThealth for cardiovascular diseases was integrated with primary health care services and evaluated in 8 villages in Malang district, East Java, Indonesia (2016-2018). It was associated with an 8.3 (95% CI –10.1 to –6.6) mmHg reduction in BP [21]. The strategy has been adopted by the Malang district government for scale-up and integration with existing electronic medical record system (e-Puskesmas) in over 390 villages (2020-2023) and is being evaluated as part of the Global Alliance for Chronic Diseases scale-up funding round. Digital technologies, including affordable and innovative solutions, allow patients and health care providers to make evidence-based decisions.

The SMARThealth program [20] has been adapted for optimal management of patients with diabetes and kidney disease in the context of Thailand’s health system and comprises the following elements. First, a platform for community nurses to assess the disease risk using a clinical decision support system (CDSS) app on an Android tablet device. The app allows community nurses to collect essential health-related information from patients, inform them of their risk status, provide lifestyle advice relating to physical activity, diet and tobacco and alcohol, and refer high-risk patients to the specialist doctor at the district hospital. In addition, the app provides decision support to community nurses for providing guideline-based recommendations and adjusting medication prescriptions during follow-up. Second, a 2-day training induction and ongoing support from the research team for community nurses. Third, data collected by the community nurses are asynchronously uploaded to a shared electronic medical record (OpenMRS) via the Sana Mobile Dispatch Server and stored on a centralized server in Thailand. Finally, availability of a central support team to visit the community nurses periodically and provide support such as re-training, co-ordinating, and solving IT issues. The quality of intervention is ensured by supervisor field visits.

### Hypothesis

The primary study hypothesis is that compared with usual care, adapting, and implementing a multifactorial intervention (SMARThealth) that strengthens the existing primary health care system and addresses known constraints for diabetes management in rural Thailand will improve blood glucose levels and thereby reduce renal complications among adults with type 2 diabetes.

### Main Study Objectives

The study objectives are to (1) adapt, validate, and integrate the SMARThealth Diabetes program for improving the management of type 2 diabetes mellitus at the primary health care level; and (2) determine if the SMARThealth Diabetes program is feasible for implementation and acceptable in the rural communities of Thailand.

## Methods

### Study Design

**Figure 1:**
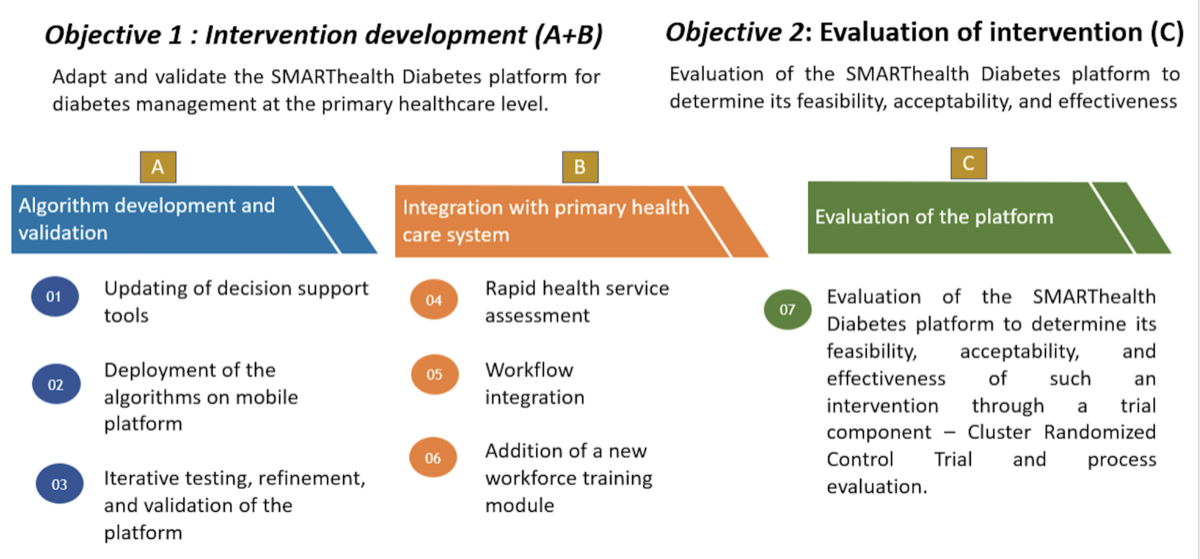
SMARThealth Diabetes—study design and objectives.

The overall study design is presented in Figure 1.

### Objective 1: Intervention Development

#### Algorithm Development and Validation

##### Updating of Decision Support Tools

The existing global guideline-based algorithms [22] for the screening and management of diabetes were updated with recommendations consistent with Thai guidelines [23]. These included evidence-based guidance on monitoring BP and blood glucose, lifestyle changes (smoking cessation, weight loss, improved diet, exercise, alcohol, and sodium restriction), and adherence to medical treatments, including renin angiotensin aldosterone system blockade. The draft algorithm underwent expert review by leading diabetes and kidney disease specialists to assess consistency with global and local guidelines. Drugs for diabetes, hypertension, and hyperlipidemia were identified according to their availability at the SDHOs. The recommendations were converted into “pseudo-code” rules and developed into detailed software specifications. After programming, the algorithms underwent independent clinical and statistical validation using established methods [20].

##### Deployment of the Algorithms on the Mobile Platform

The algorithms were integrated and deployed using the existing Java-based SMARThealth platform, which can be accessed directly on low-cost Android devices. The existing platform was modified to accommodate additional inputs and outputs based on the updated algorithm. The recorded input information was ensured to be securely transferred (synchronous or asynchronous) to a shared electronic record located on a central server in Thailand. Output providing referral and monitoring recommendations for the nurses are generated based on the parameters entered. Nurses can adjust medications through the platform, and if a referral to a higher center is needed, referral advice with details of the referral center is also provided.

##### Iterative Testing, Refinement, and Validation of the Platform

The platform was rigorously validated in 2 steps. The first is to ensure that the local guidelines are correctly interpreted by the algorithm, and the second is to ensure that it is accurately deployed in the Java-based mobile platform. The outputs from the SMARThealth platform of about 100 patients were run through 2 clinicians to estimate the accuracy of the recommendations. Minor changes, as suggested by clinicians were considered, and the algorithms were modified till both clinicians were satisfied with the final recommendations.

#### Integration of the SMARThealth Platform With the Primary Health Care System in Thailand

##### Rapid Health Service Assessment

An audit of health service capacity was conducted using qualitative methods, including in-depth interviews and focus group discussions (FGDs) with community members, health care providers and district administrators in five primary catchments at all levels of health care. The interviews were conducted in local language (Thai) and led by a moderator trained in interview and group facilitation techniques. A total of 20 in-depth interviews and 2 FGDs were conducted with a total sample of 36 participants in 5 primary health care catchment areas, till no new information was obtained from the interviews. This assessment identified barriers and facilitators for the multifaceted system intervention implementation and identified modifications that will be required to maximize the likelihood of successful integration and implementation. Findings of the rapid health service assessment will be published separately.

##### Workflow Integration

Through the inputs from the health service assessment and ongoing discussions with nurses from SDHOs and doctors at health facilities, the SMARThealth platform was adapted to suit the existing workflow of the health offices. For example, the paper registration forms were digitized and integrated with the platform, the existing printers at the SDHOs are used to print treatment advice and referral cards, available laboratory records at SDHO are used for input into the platform, and mobile communication of doctors and nurses are retained through use of this platform.

##### Addition of a New Workforce Training Module

A SMARThealth Diabetes training program was developed. Inputs from the rapid health system assessment helped in the planning of the training program in terms of location of training, content, and timings. The training package is delivered in an initial workshop format and continuously reinforced through the program’s digital platform. The training program delivery is supported by training manuals to nurses and doctors to ensure local relevance.

### Objective 2: Evaluation of the Platform

#### Study Design

The intervention will be evaluated using a pragmatic, type 2 hybrid effectiveness or implementation, parallel-group cluster randomized controlled trial of 12 months duration. A total of 51 SDHOs will be randomized 1:1 into intervention and control arms. As the intervention is directed at the health system involving the nurses, doctors, and other staff working as teams, effectiveness is best evaluated through cluster randomized controlled trial with clustering at the SDHO level.

#### Study Setting

The study is conducted in rural communities in Kamphaeng Phet province in Thailand through the involvement of SDHOs selected in consultancy with the ministry. To be eligible, the SDHO personnel must be willing to participate in the study, have interest in using digital technology, and not participating in any competing research study.

### Eligibility Criteria

#### Inclusion Criteria

Aligning with the Thai public health screening policies, and in discussion with MOPH, patients attending SDHOs will be eligible to participate if they are (1) aged 30 to 70 years; (2) known cases of type 2 diabetes currently on oral hypoglycemic medications or identified to have diabetes in the initial screening by the community nurses; (3) have CKD-EPI eGFR of > 60 mL/min/1.73 m² [24]; and (4) undergoing treatment at the SDHOs by a community nurse.

#### Exclusion Criteria

Participants with the following characteristics will not be eligible: (1) individuals who decline participation; (2) patients with a diagnosis of nondiabetic kidney disease (for example, ADPKD, lupus nephritis, obstructive uropathy, renal stone, nephrotic syndrome, or primary glomerular disease); (3) patients with expected life expectancy of less than 2 years; (4) patients with AIDS; (5) patients with known pregnancy; and (6) patients having communication problems, for example, migrant workers and people with dementia.

### Informed Consent

Consent will be obtained based on a professional-cluster design approach proposed by Hemming and Eldridge [25]. Consent will be collected at three levels: (1) at the cluster level —verbal approval from each SDHO to participate in the trial; (2) from the health workers—verbal approval from nurses and doctors to participate in the study and provide information during and after the study; and (3) from individual participants—consent from patients with diabetes interested in participating in the study for data collection during the study.

### Intervention Group

SDHOs randomized to the intervention group will receive the SMARThealth Diabetes program described earlier.

First, a workforce restructuring and training program to increase the involvement of community nurses in routine aspects of diabetes and kidney disease care. Using the module developed in objective 1, training will be provided to community nurses to promote awareness of lifestyle determinants, use of the CDSS to record risk factor information from the laboratory records available at the health center, guidance in the interpretation of the CDSS output, and processes to refer high-risk individuals to the district hospitals and training to monitor and promote adherence to prescribed medications. The training will be imparted for 2 days. After training and certification, community nurses will each be provided with a 7-inch tablet preloaded with the SMARThealth software. Second, the Thai CDSS will incorporate components related to referral, follow-up, and personalized recommendations about blood glucose control, BP lowering, pharmacotherapy including ACE inhibition, statin, and eye and foot care. Third, continuous performance monitoring. The performance of community health nurses will be monitored using standard, automated reports generated in real time and based on certain key performance indicators (eg, the proportion of individuals with diabetes on recommended combination treatment). These reports will be provided to health administrators in each district.

### Criteria for Discontinuing or Modifying Allocated Interventions

There will be no predefined criteria for stopping the allocated intervention. In case new national clinical guidelines are published during the trial, this may justify modification of the content of the intervention without changing the form of the intervention. The decision for modification will be made by the steering committee.

Participants may withdraw from the trial at any time. Withdrawal will not negatively affect their ability to access usual care from the SDHO.

### Control Group

SDHOs randomized to the control group will continue with usual practice, without access to the training and support package, the CDSS and associated tools.

### Outcomes

#### Primary Outcome

The primary outcome will be the difference in change in mean hemoglobin A_1c_ (HbA_1c_) measured at the SDHO between randomization and 12 months from randomization, between the intervention and control clusters. HbA_1c_ has been chosen as the primary outcome as it is recommended by local clinical guidelines as a treatment target.

#### Secondary Outcomes

Secondary outcomes will include the difference in change in albuminuria status; change in eGFR, systolic blood pressure, and low-density lipoprotein cholesterol level; change in the proportion of patients with HbA_1c_<7%, and patients with systolic blood pressure<140 mm Hg; measured at randomization and 12 months from randomization, between the intervention and control clusters.

### Timeline

SDHOs will be recruited and randomized before individual participant recruitment commences. After randomization, community nurses in the intervention clusters will receive the training program (as described earlier). Eligible patients with diabetes registered within the SDHOs will be invited by the community nurses to participate in this study. After obtaining written informed consent, demographic and additional data, baseline parameters will be collected using previously validated, structured questionnaires. At the end of the study (12 months), data and biological samples will again be collected at the laboratory in SDHOs by trained laboratory technicians, along with completing the endline questionnaires by community nurses to allow analyses for all outcomes.

### Statistical Considerations

**Figure 2:**
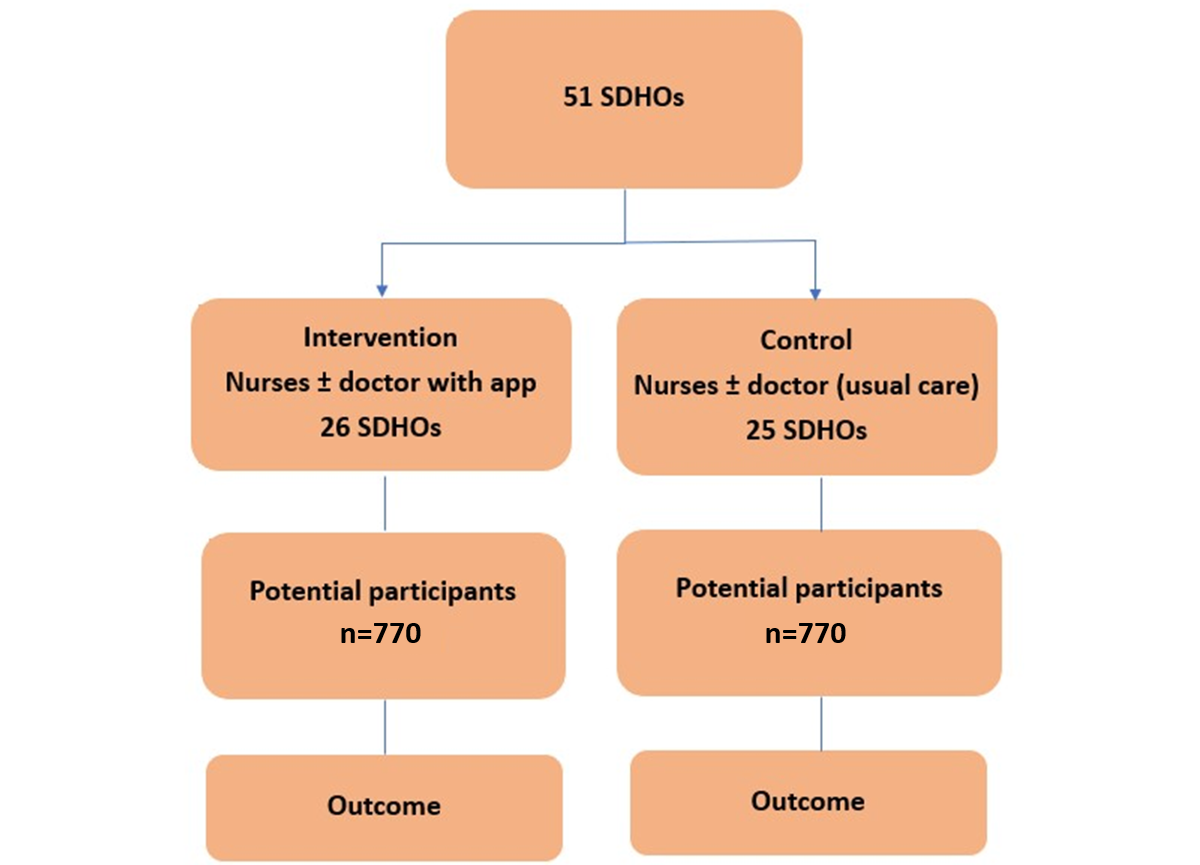
Randomization and sampling based on the SDHOs. SDHO: subdistrict health office.

Randomization of 51 SDHOs with an average cluster size of 40 individuals with diabetes at baseline will provide 90% power (2α=0.05) to detect an absolute mean HbA_1c_ difference of 0.5%. A change of 0.5% in HbA_1c_ is considered clinically meaningful [26]. Intracluster correlation coefficients of 0.05 for HbA_1c_ are assumed. By assuming a 10% dropout rate in our study, the sample size calculation indicated that a total of 1540 participants will be required to be recruited in both arms, with 770 in each group (Figure 2).

The analysis of change in HbA_1c_ between baseline and the end of study (primary end point) will be performed using a linear mixed model including treatment arm and baseline HbA_1c_ as fixed effects as well as SDHO as a random effect to account for clustering of individuals by village. In case of baseline imbalances between the 2 arms at the cluster or individual level, unbalanced characteristics will be added to the models as a sensitivity analysis. The same approach will be applied for continuous secondary end points.

### Data Collection and Management

Data collection will commence at baseline (0 months; randomization) and will be conducted quarterly at 3, 6, 9, and 12 months after randomization by community nurses. All data will be captured on tablet devices using the SMARThealth app adapted for the clinical research forms (CRFs). The CRFs collect information on anthropometric measurements, medical history, and current medication for hypertension, diabetes, and hyperlipidemia, in addition to laboratory test results. These CRFs will be completed at months 0, 3, 6, 9, and 12. The primary outcome variable (HbA_1c_) and other laboratory tests will only be recorded at months 0, 6, and 12. Data will be deidentified, saved, and stored in a secure server located in Bangkok, Thailand. Published, validated questionnaires are used in the CRFs and tested using the SMARThealth platform before data collection. About 10% of all CRFs will be checked for accuracy against source data by the project team.

SMARThealth platform will be used for digitally collecting all baseline and outcome data. Data access will be restricted to delegated study staff and will require multifactor identification with a digital log kept of all logins. The project manager and data managers will be responsible for data quality. The project manager will ensure quality by interacting periodically with community nurses and checking for completeness of data, and data validation.

### Confidentiality

Participant confidentiality will be maintained throughout the study. Each participant in the study will be assigned a 10-digit unique alphanumeric patient ID. The first 2 digits are the district code, the following 5 digits are the SDHO code, and the last 3 digits indicate the individual patient ID. This unique ID ensures that their personal identifiers remain anonymized throughout the research process.

Our data management system employs 3 robust security layers to safeguard patient data. First, password protection is implemented to regulate access, preventing unauthorized usage by individuals. Second, local data protection measures, including encrypted data communication and role-based access, are implemented to ensure the security of the locally stored data at the device level. Finally, at the server level, additional measures such as firewall protection, reliable maintenance, monitoring, and support services are implemented to safeguard the database.

### Process Evaluation

A detailed awareness of local contextual factors will be undertaken to understand the impact of SMARThealth and barriers to its implementation and scale-up. The process evaluation will be designed in line with recommendations by the UK Medical Research Council in its “guidelines for developing and evaluating complex interventions” [27,28]. Evaluation data, data collected during intervention delivery, and data collected through qualitative methods will be used to investigate the process evaluation framework that includes identifying factors that promote or inhibit the implementation and maintenance of the program [29]. A maximum variation sampling technique will ensure diverse opinions are captured from participants and health care workers. The process evaluation will be informed by normalization process theory (NPT) [30] to assess the extent to which the new system fits within the normal processes of the current service provision in the villages and SDHOs. The 4 main concepts of NPT namely, coherence, cognitive participation, collective action, and reflective monitoring will be identified in the process of implementation of the SMARThealth diabetes program in the intervention SDHOs.

Key evaluation areas that the study will explore are: (1) how the health workers use the intervention, (2) what effects the intervention might have on doctor practices, and (3) participant experiences of receiving the intervention.

Qualitative data will be collected using semistructured, in-depth interviews and focused group discussions [31]. NPT will inform the semistructured interview guides and will also be used as a framework for analyzing the data. All interviews and group discussions will be audio-recorded, and then transcribed verbatim. The qualitative data will be thematically analyzed (from codes to categories and finally themes) to identify and interpret patterns and themes within the data. Data will be analyzed contemporaneously, and data collection will be stopped when thematic saturation is reached. Two researchers who are trained in qualitative methodology will be involved in coding the interview and FGD transcripts. In the event of a disagreement, a consensus meeting will be held with the principal investigator to reach an agreement on interpretation of the data. Intercoder reliability will be reported using qualitative assessments of agreement and consistency among the coders using processes described by O’Connor and Joffe [32]. Process evaluation data will be analyzed independently of the outcome evaluation data and then the 2 sets of data will be combined and triangulated to increase the reliability of the results.

### Ethical Considerations

This program will be conducted, evaluated, and reported in compliance with all local regulatory requirements in Thailand. Ethical approval has been obtained from the Ethical Review Committee for Research in Human Subjects at MOPH in Thailand (25/2562) and the Oxford Tropical Ethics Research Committee (21/19).

All potential participants for both the qualitative and quantitative component of the study will be provided with a participant information sheet prior to their recruitment into this study.

Participation in the study is voluntary, and no financial compensation will be provided to the participants. Data collected using the SMARThealth Diabetes platform will be stored securely in a central server located in Bangkok, Thailand. Personal identifiable information will be stored securely; electronic data will be deidentified and analyzed. Findings will be written up as means and published without identifying information. Electronic audio files from interviews will be stored electronically on a local network server at the researcher’s organization, under both firewall and password protection. Access will be limited to study investigators. All the data collected in the study will be managed by the central research team. The data collection will be routinely monitored for completeness and quality control checks will be introduced. The physical documents will be stored securely and only accessible by the study researcher.

## Results

The study was funded by the Medical Research Council, United Kingdom in April 2018 with additional funding received from Medical Research Council and Imperial College London in September 2022. We had to delay the recruitment of the participants into the trial between the period 2020-2021 due to the unprecedented COVID-19 pandemic. As of May 2024, we have enrolled 1599 patients with type 2 diabetes, who are managed and followed up by community nurses at 51 SDHOs. Final data analysis and results are expected to be published in the first quarter of 2025.

## Discussion

### Expected Findings

This study will address the growing epidemic of diabetes leading to CKD and kidney failure in Thailand. To our knowledge, the SMARThealth Diabetes study is the first randomized trial to use technology as a health system intervention to support the delivery of optimal management of diabetes at a primary health care level in Thailand. This intervention is designed to address several gaps in the treatment of diabetes and kidney disease, including inadequate workforce, disproportionate focus on physician-centric care models not aligned with the values and preferences of communities in which they are implemented, variation in the quality of care, and its high cost.

The SMARThealth Diabetes intervention relies on primary health care workforce reengineering and an electronic decision support with an emphasis on minimizing variations on quality of care. These components are embedded in the public health care system, enhancing the adaptation and sustainability of the intervention. Similar primary health care interventions have already been successfully implemented in primary health care settings in Indonesia and India for detection, prevention, and management of cardiovascular diseases [21,33]. Hence, there is a need to extend the platform to similar primary health care settings in other low- and middle-income countries. The evidence generated through this study will have substantial potential to inform policymakers and system planners to include high-quality primary health care for common NCD conditions.

### Strengths and Limitations of the Study

There are many strengths in this study, including the use of a novel multifaceted mobile health strategy to engage communities, physicians as well as nonphysician health care providers in learning how to cope with diabetes and, as a result, strengthen the overall health system. Additionally, a comprehensive process evaluation will allow the assessment of the feasibility, scalability, and sustainability of such a strategy in the local health care system.

The main limitation is that it is conducted in selected rural areas, which might not represent the wider population in other districts and provinces in Thailand. However, integrating the intervention into the health system, which is similar throughout the country will help to learn from this project and support future scale-up if successful. Further, although this project will be deployed in rural Thailand, the components of the intervention are generic, and this study will generate important evidence that will inform the adaptation of similar interventions in other health care settings. Aligning to the study design, the study will need to collect information on HbA_1c_ and other laboratory tests from participants in both arms leading to better than usual care in the control arm. We have streamlined the timing of the data collection to the routine follow-up visit of patients in the control arm. Still, we need to acknowledge there could be additional communication with these patients to ensure follow-up visits are happening as per the need of the study.

### Conclusions

In conclusion, we present a study protocol for a pragmatic, parallel group cluster randomized hybrid effectiveness or implementation trial to test the SMARThealth Diabetes platform in rural Thailand. The study aligns with guidelines developed by the Medical Research Council for developing and evaluating complex interventions. The platform will support the delivery of optimal management of diabetes at a primary health care level and we hope this trial will demonstrate the effectiveness of the platform in addressing the gaps in the treatment of diabetes and kidney diseases.

## Abbreviations

BP: blood pressure
CDSS: clinical decision support system
CKD: chronic kidney disease
CRF: clinical research form
eGFR: estimated glomerular filtration rate
FGD: focus group discussion
HbA1c: hemoglobin A1c
MOPH: Ministry of Public Health
NCD: noncommunicable disease
NPT: normalization process theory
SDHO: subdistrict health office

## Data Availability

The data sets generated or analyzed during this study will not be publicly available in accordance with the data management plan but will be made available from the corresponding author on reasonable request.
